# The radiological findings of COVID-19

**DOI:** 10.3906/sag-2106-203

**Published:** 2021-12-17

**Authors:** Alper KARACAN, Yakup Ersel AKSOY, Mehmet Halil ÖZTÜRK*

**Affiliations:** 1 Department of Radiology, Faculty of Medicine, Sakarya University, Sakarya Turkey

**Keywords:** COVID-19, imaging, computed tomography, signs, extrapulmonary involvement

## Abstract

**Background/aim:**

Available information on the radiological findings of the 2019 novel coronavirus disease (COVID-19) is constantly updated. Ground glass opacities (GGOs) and consolidation with bilateral and peripheral distribution have been reported as the most common CT findings, but less typical features can also be identified. According to the reported studies, SARS-CoV-2 infection is not limited to the respiratory system, and it can also affect other organs. Renal dysfunction, gastrointestinal complications, liver dysfunction, cardiac manifestations, and neurological abnormalities are among the reported extrapulmonary features. This review aims to provide updated information for radiologists and all clinicians to better understand the radiological manifestations of COVID-19.

**Materials and methods:**

Radiological findings observed in SARS-CoV-2 virus infections were explored in detail in PubMed and Google Scholar databases.

**Results:**

The typical pulmonary manifestations of COVID-19 pneumonia were determined as GGOs and accompanying consolidations that primarily involve the periphery of the bilateral lower lobes. The most common extrapulmonary findings were increased resistance to flow in the kidneys, thickening of vascular walls, fatty liver, pancreas, and heart inflammation findings. However, these findings were not specific and significantly overlapped those caused by other viral diseases, and therefore alternative diagnoses should be considered in patients with negative diagnostic tests.

**Conclusion:**

Radiological imaging plays a supportive role in the care of patients with COVID-19. Both clinicians and radiologists need to know associated pulmonary and extrapulmonary findings and imaging features to help diagnose and manage the possible complications of the disease at an early stage. They should also be familiar with CT findings in patients with COVID-19 since the disease can be incidentally detected during imaging performed with other indications.

## 1. Introduction

Coronavirus disease (COVID-19) is an infectious disease caused by severe acute respiratory syndrome coronavirus 2 (SARS-CoV-2). After COVID-19 was first reported in Wuhan, China, in December 2019, it spread rapidly worldwide and was declared a pandemic by the World Health Organization (WHO) on March 12, 2020 [1]. The SARS-CoV-2 virus has had devastating effects on human life, health systems, and economies. SARS-CoV-2 is a beta-coronavirus that infected more than 167 million people as of June 2021, resulting in more than 3 million deaths worldwide. A significant proportion of those with COVID-19 infection remain asymptomatic (40%–50%) or show relatively mild symptoms (40%) [2]. COVID-19 disease caused by SARS-CoV-2 typically presents with weakness, fever, cough, and sore throat but can progress to pneumonia, bronchitis, and acute respiratory distress syndrome (ARDS). However, since none of these symptoms are specific to COVID-19, and the disease can rapidly progress to severe pneumonia, multiple organ involvement including renal failure, vascular thrombosis, ischemia, respiratory distress, and gastrointestinal symptoms, there is an urgent need for rapid diagnostic tests [3]. Although the reverse transcriptase-polymerase chain reaction (RT-PCR) test for viral nucleic acids is the gold standard in the diagnosis of COVID-19, radiological imaging, especially computed tomography (CT) has become increasingly important in diagnosis due to the false-negative results of the RT-PCR test [4]. Studies have shown that the sensitivity of CT is between 60% and 98% [5,6]. This range is relatively higher compared to the RT-PCR test (60%–70%), but its specificity (25%–53%) is low. The positive and negative predictive values of chest CT for COVID-19 are estimated at 92% and 42%, respectively. The relatively low negative predictive value reduces the value of CT as a screening test for COVID-19 in the early stages of the disease [7]. In addition, considering that it contains ionizing radiation, CT should be used as a problem-solving method in patients with a negative RT-PCR test rather than as a screening method [8]. Imaging plays an essential role in the management of COVID-19. In this study, we aimed to review the areas of use and imaging findings of various imaging methods, such as chest radiographs, CT, and ultrasonography in the diagnosis and treatment of COVID-19. 

## 2. Thoracic imaging in COVID-19

### 2.1. Chest radiography 

Chest radiography is often the first imaging modality of choice for patients with known or suspected COVID-19 pneumonia. In the literature, the diagnostic value of chest X-ray in COVID-19 pneumonia is reported to vary between 30% and 60%, which is relatively low. Although it is possible to detect some abnormalities in the presence of viral pneumonia on chest X-ray, normal findings obtained from this modality do not exclude the disease. Moreover, if the disease progresses, the possibility of an abnormal chest radiograph increases [7,9,10].

Chest radiography often does not show any abnormality in the early stage of COVID-19 infection. Therefore, radiography has not been recommended as a first-line method for the investigation of chest abnormalities in patients with suspected COVID-19 [11]. However, in mild COVID-19 conditions, chest radiography may demonstrate local irregular radiopacities in the outer parts of the lungs and the subpleural region [12]. COVID-19 lung involvement occurs in bilateral involvement of the peripheral and lower lobe basal segments [13]. In severe cases, there is a widespread, sometimes patchy, multiple consolidation with a small amount of pleural effusion. In critically ill patients, areas of consolidation covering the entire lung are referred to as white lung [14]. 

In conclusion, the diagnostic contribution of chest radiography is lower than that of chest CT, and noncontrast chest CT should be considered for the early diagnosis of viral disease in suspected patients with normal chest radiography findings [10].

### 2.2. Ultrasonography (USG)

USG is one of the most commonly used imaging methods in clinical practice and allows for the systematic and rapid examination of multiple organs at the bedside without using radiation. The general opinion is that USG cannot detect lung lesions; however, many lung pathologies can be imaged with this modality. The main signs of lung ultrasound in COVID-19 can be defined as A-lines, B-lines, subpleural consolidation, and lung deviation signs [15]. In COVID-19-infected cases, the occurrence of pulmonary lesions predominantly in the peripheral regions of the lung and the subpleural areas of both lungs facilitates sonographic imaging [16]. However, deeply located lung lesions cannot be detected on USG, and it is difficult to use this imaging method in the differential diagnosis of pneumonia. USG is not recommended in the diagnosis of mild cases but it can be considered as an additional pulmonary and cardiac evaluation method, especially in critically ill patients who should not be moved and for whom a CT examination is not possible [17].

USG is particularly important in examining critically ill patients and determining treatments, particularly hemodynamic therapy. It is helpful in functional imaging rather than anatomical imaging, and its applications can be summarized as follows [18,19]: 

First, USG is an imaging method that offers the rapid evaluation of the disease. An increase in the number of B-lines and the area of ​​consolidation may indicate lung disease progression [20]. Second, respiratory support is required in severe and critical cases, but the time point for the transition from noninvasive ventilation to invasive mechanical ventilation is essential [21]. Lung USG showing progressive diffuse B-lines and significantly increased diaphragm activity may indicate severe lung damage. When mechanical ventilation cannot improve oxygenation, extracorporeal membrane oxygenation (ECMO) may be a treatment option. USG provides visual management in the ECMO process [22]. Third, echocardiography (cardiac ultrasound) can be used to evaluate circulatory functions. The most frequently reported complication in critically ill patients with COVID-19 is ARDS, followed by acute heart damage and secondary infection [23]. Echocardiography can also quickly assess hemodynamics and determine whether there are segmental wall motion abnormalities, stress-induced cardiomyopathy, or acute right heart dysfunction that may guide treatment [24,25]. Finally, coagulation abnormalities have been reported in approximately 20% of critically ill COVID-19 cases [23,26]. Sudden worsening of the condition often suggests a state of hypercoagulation with a dramatic increase in the D-dimer level [27]. Asymmetrical pain or swelling may indicate the risk of lower extremity deep vein thrombosis (DVT) for patients staying in bed for more than three days. For patients with central venous catheters, local swelling of catheterized limbs may indicate the risk of upper limb DVT. In all these cases, vascular structures can be evaluated with color Doppler USG [28]. In this way, effective and timely interventions can be provided for patients at high risk of embolism [12].

The main limitation of USG is that in general it has poor specificity, and its findings overlap with those of other pneumonia etiologies or incidental chronic findings (e.g., chronic heart failure or pulmonary fibrosis)[29].

### 2.3. Chest CT

CT is sensitive the early signs of COVID-19 and changes that may not be seen on chest radiography [30]. It is essential to know that in 50% of patients with COVID-19 symptoms, an abnormal chest CT finding may be expected in the first days after symptoms appear [13]. On the contrary, lung changes due to COVID-19 can be monitored on CT in patients with no symptoms and are usually seen incidentally.

Chest CT can have characteristic features of COVID-19 during active infection, and therefore it can help monitor the course of the disease and provide timely treatment. The hallmark of COVID-19 is the bilateral presence of patchy GGOs that can merge into dense, consolidative lesions with a predominantly peripheral distribution in the subpleural areas, as well as bronchovascular bundles [31,32]. As the disease progresses, the number of lesions can rapidly increase and extend into central regions [33]. During the recovery of the disease, lesions gradually regress over two weeks, during which time fibrotic changes may occur [34]. In addition to GGOs and consolidations, many other CT findings, such as interstitial thickening, halo sign, inverted halo sign, and airway-vascular changes can be seen in COVID-19 pneumonia. These findings and distribution patterns may help distinguish COVID-19 pneumonia from other types of pneumonia [35].

#### 2.3.1. Ground glass opacities (GGOs)

**Figure 1:**
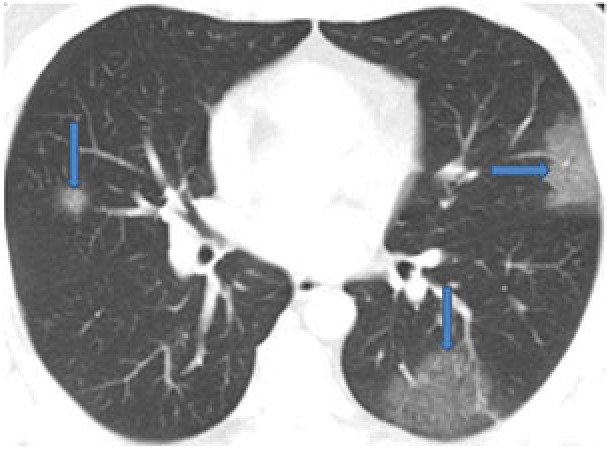
A 35-year-old male COVID-19 patient presenting fever and headache for four days. CT scan shows pure ground-glass opacities in bilateral multilobe (arrows).

GGOs are nonspecific findings defined as hazy lung opacities that do not cover the underlying vascular or bronchial boundaries and are assumed to be related to partial airspace filling or interstitial thickening (Figure 1) [36]. The predominant CT pattern in COVID-19 is bilateral GGOs associated with consolidations, but findings may differ from patient to patient or depending on the stage of the disease [31,32]. It has been reported that GGOs are seen at a rate of 50%–95%, and they are isolated in 50% of cases and accompanied by consolidations in 30%–40% [37–39].

#### 2.3.2. Consolidations

Consolidations result from the complete displacement of alveolar air spaces by pathological fluids or cells, leading to an increase in parenchymal density that obscures the underlying vessels and bronchial walls [36]. Consolidations have been reported in 20%–63% of cases and may be multifocal, irregular, or segmental with subpleural or peribronchovascular distribution [40,41]. Consolidations occur with the progression of the disease within two weeks after the onset of the disease and often accompany GGOs [42].

#### 2.3.3. Reticular opacity

**Figure 2:**
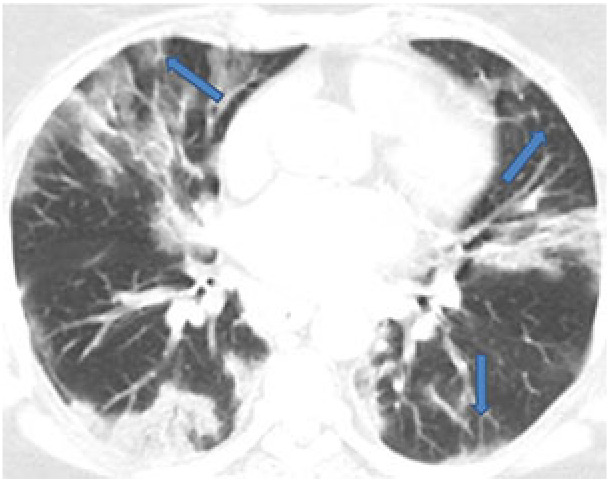
A 66-year-old male COVID-19 patient presenting fever with cough for seven days. Multiple ground-glass opacities and consolidation with a thickened intralobular and interlobular septum (arrows).

The reticular pattern consists of a complex network of linear opacities associated with interlobular and intralobular septal thickening and is caused by lymphocyte infiltration (Figure 2) [36,43,44]. With an incidence of 27%, it is the third most common finding in COVID-19 after GGOs and consolidations [40,42,45], and its incidence increases over the course of the disease [42].

#### 2.3.4. Crazy paving pattern

This pattern is defined as the presence of GGOs with overlapping interlobular septal thickening, giving the appearance of irregular cobblestones and may be associated with alveolar edema combined with the inflammation of interstitial structures [36]. In COVID-19, this finding is less frequent than GGOs and consolidations alone [34]. However, this sign has been reported in 5%–36% of patients and may be considered an indication of progression or the highest stage of the disease when accompanied by GGOs and consolidations [41].

#### 2.3.5. Air bronchograms

**Figure 3:**
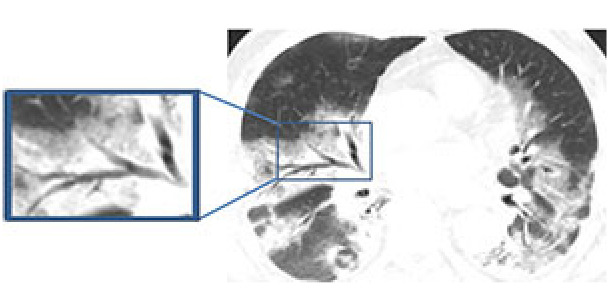
A 66-year-old female COVID-19 patient presenting fever with cough for nine days. The air bronchogram sign in right lower lobe (frame).

Air bronchograms, in which air-filled bronchi can be seen in a highly attenuated parenchymal background, have been recorded in series of patients affected by COVID-19 (Figure 3) [36,45]. However, some autopsy studies indicate that the bronchi are actually filled with gelatinous mucus plugs; therefore, the term bronchiolectasis may be more appropriate than air bronchogram because the bronchi are not filled with air and are associated with mild bronchial dilatation [46]. Similar studies have also emphasized that the high viscosity of mucus can cause bronchiolar damage, and thus lead to bronchiolectasis and characteristic dry cough in patients with COVID-19 [35].

#### 2.3.6. Airway changes

Airway changes reported in association with COVID-19 occur as a result of bronchiectasis in some patients and bronchial wall thickening in 10%–20% of cases [5,40,41]. The pathological mechanism is bronchial obstruction and inflammatory damage of the bronchial wall, both of which lead to the destruction of the bronchial wall structure and development of fibrosis, and consequently bronchiectasis [36]. In addition, the percentage of bronchial wall thickening has been determined to be significantly higher in patients with critical COVID-19 [41].

#### 2.3.7. Pleural changes

The most common pleural change in patients with COVID-19 is not pleural effusion (5%) but pleural thickening (32%) [42]. The presence of pleural thickening was confirmed by autopsy findings [46]. The presence of pleural effusion has been suggested to be a poor prognostic factor [41,42].

#### 2.3.8. Subpleural lines

**Figure 4:**
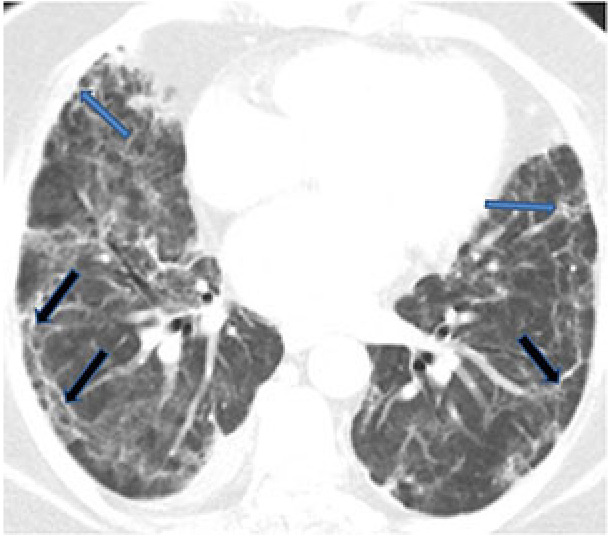
A 49-year-old female COVID-19 patient presenting chest pain for 14 days. Subpleural lines (black arrows) with thickened intralobular and interlobular septums (blue arrows).

Subpleural lines refer to thin curvilinear opacities of approximately 1–3 mm in thickness, located in the subpleural region and distributed parallel to the pleural surface (Figure 4). They are a nonspecific sign seen in 33% of COVID-19 cases, but they may also result from atelectasis, pulmonary edema, and fibrosis [47]. In addition, subpleural lines may be associated with the predominantly peripheral location of parenchymal changes in COVID-19 [48].

#### 2.3.9. Fibrosis

Fibrotic lesions may occur in patients with the proliferative disease and those recovering from chronic pulmonary inflammation due to the replacement of cellular components by scar tissue [49]. Fibrosis is usually seen at two to three weeks after the onset of COVID-19 when interstitial changes begin to occur [50]. Some studies have suggested that the presence of fibrosis indicates advanced COVID-19, which can lead to bronchial deformation. However, the general belief is that fibrosis is a part of the recovery process [51,52].

#### 2.3.10. Nodules

A pulmonary nodule is defined as a round or irregular parenchymal opacity of less than 3 cm in diameter and often associated with the presence of viral pneumonia [36,52]. Multiple solid nodules with irregular contours have been found in 3%–13% of patients with COVID-19, sometimes accompanied by a halo sign [34,53].

#### 2.3.11. Halo sign

This is described as a nodule or mass surrounded by GGOs [36]. The pathological mechanism underlying this sign remains unclear. However, apart from perilesional hemorrhage being a possible sign of viral infections and organized pneumonia, the halo sign has also been associated with angioinvasive fungal infections or hypervascular metastases [54,55].

#### 2.3.12. Reversed halo sign

**Figure 5:**
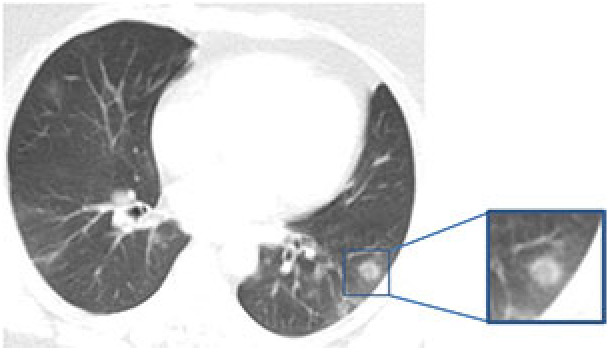
A 48-year-old male COVID-19 patient presenting fever for five days CT scan shows a reversed halo sign (central ground-glass opacity surrounded by denser consolidation of crescentic shape) in left lower lobe (frame).

The presence of a ring-like consolidation zone with an overlapping circular GGO is defined as an inverted halo sign (Figure 5) [36], which has been reported to be associated with COVID-19. It is considered as a healing lesion with a low-density core or a new lesion that develops around a preexisting GGO [56,57]. In addition, it has been previously described in association with cryptogenic organizing pneumonia and other pulmonary infections [58,59].

#### 2.3.13. Vascular changes

The dilation of the pulmonary vessels can be caused by proinflammatory factors resulting in capillary wall damage and edema. The onset of the disease can occur in as early as one to seven days [51].

#### 2.3.14. Cavitation and air bubble sign

**Figure 6:**
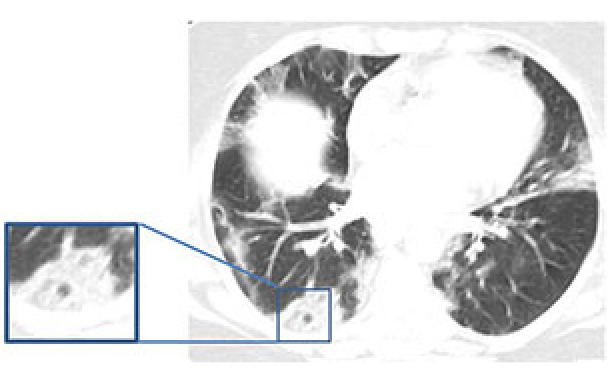
A 49-year-old male COVID-19 patient presenting fever with diarrhea for three days. The air bubble sign (frame) with GGO and consolidation in the right inferior lobe.

Cavitation is extremely rare in patients with COVID-19 [60]. The air bubble sign, defined as the small air-containing areas associated with bronchiolectasis, is also uncommon but can be seen in COVID-19 cases (Figure 6) [61].

#### 2.3.15. Lymphadenopathy

When the short-axis diameter of the mediastinal lymph node is more than 1 cm, it is defined as lymphadenopathy [36]. This finding has been reported in 4%–8% of patients with COVID-19 and considered to be an essential risk factor for severe pneumonia [40–42]. However, bacterial superinfection should be suspected when lymphadenopathies are accompanied by pleural effusion and small lung nodules [38,62,63].

#### 2.3.16. Pericardial effusion

Pericardial effusion has been reported in approximately 5% of patients with COVID-19 and assumed to be associated with the presence of severe inflammation [40,41].

### 2.4. Differential diagnosis

CT showing high sensitivity (97%) but poor specificity (25%) for the diagnosis of COVID-19 has been primarily attributed to the COVID-19 pneumonia findings overlapping those of other viral types of pneumonia, especially influenza viruses, parainfluenza virus, adenovirus, and respiratory syncytial virus [64–66]. Similar to COVID-19, most viral types of pneumonia have a predominant distribution in the posterior and peripheral parts of the lungs, affecting more than one lung lobe. However, some findings may be useful in the differential diagnosis. For example, the main feature of pneumonia caused by the respiratory syncytial virus is small centrilobular nodules and consolidation areas, usually distributed asymmetrically in the lungs. Adenovirus pneumonia on chest CT shows bilateral multifocal GGOs with irregular consolidations and may have a lobar or segmental distribution. In parainfluenza virus pneumonia, the presence of centrilobular nodules with bronchial wall thickening may help distinguish it from other viral types of pneumonia [33]. 

The radiological appearance of COVID-19 is not much different from pneumonia associated with the other two coronaviruses, SARS and MERS, possibly because they belong to the same family of *Coronaviridae*, and they have similar underlying pathological features. However, unlike the reported SARS cases, COVID-19 pneumonia shows a multifocal tendency of distribution and a peripheral distribution of GGOs in the upper lobes and a basilar or subpleural preference in the lower lobes.

The consolidation frequency and severity score of COVID-19 pneumonia are also much lower than SARS, which may explain the lower mortality rates of the former. The more peripheral distribution of COVID-19 pneumonia in the upper lobes can be used to distinguish it from MERS [67]. In contrast to COVID-19 pneumonia, SARS and MERS pneumonia usually have a single focus, and no reference to halo or reverse halo signs has been made in the literature [68]. Furthermore, unlike COVID-19, neither SARS nor MERS has been associated with the findings of lymphadenopathy, pleural effusion, or nodules [67].

The CT features of COVID-19 also show some similarities with other pulmonary pathologies, such as pulmonary edema, pulmonary hemorrhage, bronchiolitis obliterans, chronic obstructive pulmonary disease, and drug-induced lung disease [66]. In pulmonary edema, chest CT displays GGOs with central distribution, usually associated with smooth interlobular septal thickening, pleural effusion, and cardiomegaly, indicating congestive heart failure. In diffuse pulmonary hemorrhage, CT usually reveals patchy or diffuse GGOs associated with consolidations or poorly defined centrilobular opacities [69]. Air trapping is the main CT feature in bronchiolitis, while bronchial wall thickening, bronchiectasis, and centrilobular opacities can also be seen. In chronic obstructive pulmonary disease, bronchial wall thickening can be seen in addition to pulmonary emphysema. Drug-induced lung diseases are very diverse and can show various lung presentations ranging from ARDS to pulmonary fibrosis. For example, the most common chest CT features in methotrexate-induced lung disease are diffuse parenchymal opacification, reticular opacities, and centrilobular nodules with a pattern of nonspecific interstitial pneumonia [66].

Since COVID-19 can show similar clinical and radiological features with many lung pathologies, it is essential to be aware of the characteristic findings of the disease in both clinical and imaging to determine its severity, predict its prognosis, and perform a differential diagnosis.

## 3. COVID-19 and extrapulmonary radiological findings

Although the respiratory spread of COVID-19 is well documented in the literature, it has also been shown that the virus is not limited to the lungs [70]. There are many studies on the extrapulmonary involvement of COVID-19, and it is accepted that some of these involvements can seriously affect the disease prognosis [71]. It is considered that SARS-CoV-2 uses angiotensin-converting enzyme 2 (ACE2) as the cell receptor for cellular access in humans [72]. ACE2 receptor is highly expressed in the lungs, kidneys, testicles, gastrointestinal system (GIS), liver, vascular endothelial cells, and arterial smooth muscle cells [73]. Therefore, all these organs and systems with a high expression of ACE2 receptors can be considered to be the targets of SARS-CoV-2 infection [74]. 

### 3.1. Genitourinary system (GUS) involvement

Kidneys are one of the most frequently affected extrapulmonary organs in patients infected with SARS-CoV-2, especially in severe cases [75–77]. The etiology of kidney dysfunction associated with COVID-19 disease is probably multifactorial [78]. The SARS-CoV-2 virus may have a direct cytopathic effect on kidney tissue or cause damage through systemic inflammatory response syndrome, cytokine release, and sepsis, as observed in other viral diseases. Renal cell damage can cause tubular atrophy, renal interstitial fibrosis, and acute kidney injury (AKI), which is strongly associated with increased mortality and morbidity [79]. Studies conducted with patients affected by COVID-19 have shown that AKI is mainly characterized by tubular injury, increased serum creatinine and urea-nitrogen concentrations [80,81]. The severity of tubular damage is also higher in patients with severe COVID-19 than in those less affected by the disease. 

**Figure 7:**
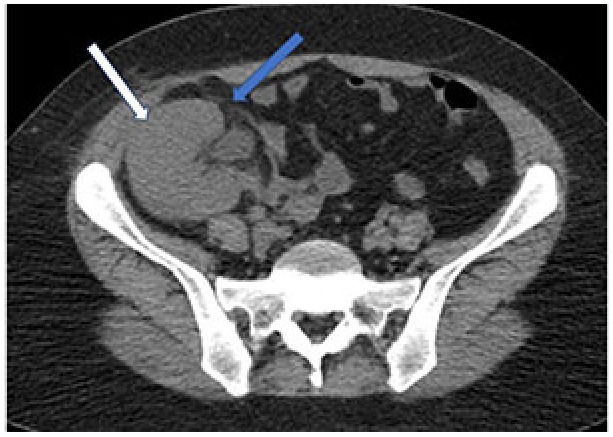
A 39-year-old female COVID-19 patient presenting with cough, fever for five days, and increased creatinine levels. CT scan shows the increased size in the transplanted kidney (white arrow) and increased density consistent with inflammation in the perirenal fatty tissue (blue arrow).

CT imaging findings in patients with AKI include inflammation and edema of the renal parenchyma characterized by lower CT attenuation values ​​than normal kidneys (Figure 7). In addition, multiple wedge-shaped parenchymal defects compatible with infarction can be seen [82,83]. Increased parenchymal echogenicity, loss of cortex-medulla separation, and thickening of the collecting system wall can also be seen in renal USG. 

A significant increase in the resistive index value (above 0.9) on the color Doppler USG examination indicates tubular damage. However, similar changes due to rejection can be seen in kidney transplant patients. In these cases, if clinical findings do not allow for a differential diagnosis, a renal biopsy is required [84–86].

Since ACE2 is produced in testicular cells, it plays a critical role in fertility. Therefore, due to potential testicular damage caused by the virus, infertility can be seen as one of the late complications of the disease. Additionally, viral orchitis may occur [87].

### 3.2. Cardiovascular system involvement

Cardiac involvement in COVID-19 disease may be due to the direct invasion of the myocardium. However, it may also be secondary to a hyperinflammatory condition, which results in atherosclerotic plaques becoming prone to rupture. As a result, endothelial dysfunction and increased procoagulant activity increase the risk of thromboembolism. In addition, 40% of patients requiring hospitalization due to COVID-19 have cardiovascular disease, which may worsen due to COVID-19 involvement [88,89].

Myocarditis is among the major complications of SARS-CoV-2 infection, accounting for 7% of deaths associated with COVID-19 [90]. In the echocardiography of patients infected with COVID-19, half of the patients show ventricular abnormalities, mainly ventricular dysfunction patterns, myocardial infarction, and signs of myocarditis [91]. Right ventricular abnormalities are common in those with the more severe disease [90]. Other findings include global or regional myocardial systolic dysfunction with or without pericardial effusion [88,89]. Right ventricular abnormalities have been reported in these patients, also reflecting the presence of severe respiratory illness, including subclinical or clinical thromboembolism [91].

CT coronary angiography can help rule out coronary artery disease in all these cases. It is also used to differentiate the pathologies of nonobstructive coronary artery myocardial infarction, cardiomyopathy, and acute myocarditis [91]. Cardiac magnetic resonance imaging (MRI) is an ideal imaging method in the diagnosis of acute myocarditis. Cardiac involvement has been detected in 78% of those who have had COVID-19 and undergone cardiac MRI, while ongoing myocardial inflammation is reported to be as high as 60% [92]. Imaging findings in these patients are abnormal T1 and T2 signals and late contrast enhancement indicating regional damage due to myocardial inflammation. The majority of high T2 signals are associated with myocardial edema in the interventricular septum and the anterior and anterolateral walls [93].

### 3.3. Peripheral vascular system involvement

Vascular involvement in COVID-19 infection may not be limited to the cardiovascular system. This disease can cause a transient state of hypercoagulation, resulting in the development of vascular thromboembolic events, such as deep vein thrombosis and pulmonary embolism if proper medication is not administered [94–96]. Although the exact pathogenesis of COVID-19-associated hypercoagulopathy remains unclear, high D-dimer levels and coagulation abnormalities have been reported in patients with the disease. These findings have been associated with a poor clinical course and higher mortality rate [97–100]. 

### 3.4. Gastrointestinal system (GIS) involvement

GIS symptoms are common in COVID-19 and may be present in up to 26% of patients. GIS symptoms usually occur one to two days after pulmonary symptoms and include anorexia (26.8%), nausea and vomiting (10.2%), diarrhea (12.5%), and abdominal pain (9.2%) [101,102]. GIS symptoms can sometimes be seen at an early stage of the disease, preceding the other typical symptoms of COVID-19 [103]. 

The spectrum of cross-sectional imaging findings of intestinal pathologies in patients with COVID-19 ranges from inflammation to ischemia and necrosis. Imaging findings include intestinal wall thickening, contrast enhancement changes in the intestinal wall, ileus, pneumatosis, and gas in the portal venous system [101,104–106].

### 3.5. Hepatobiliary system involvement

**Figure 8:**
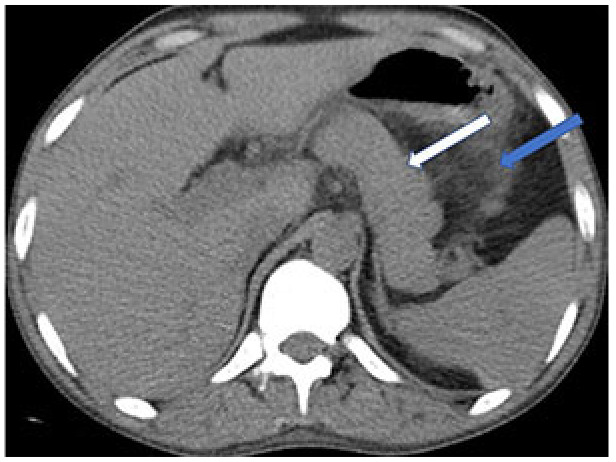
A 30-year-old male COVID-19 patient presenting with abdominal pain. CT scan shows volume increase in the pancreas (white arrow) and increased density in the surrounding fatty tissue (blue arrow), consistent with pancreatitis.

COVID-19 involvement of the liver, gall bladder, pancreas, and spleen has also been described. In liver injury associated with SARS-CoV-2, increased parenchymal echogenicity and decreased parenchymal density can be seen on the CT examination due to hepatic steatosis [104]. Infarcts may occur in the spleen, which can be visualized as wedge-shaped, low-density areas on CT [107]. Examples of pathologies in the gall bladder and tracts of COVID-19 cases are distension of the gallbladder, thickening of the bladder wall, and sludge formation without intraluminal stones [108]. Concerning the pancreas, an increase in the pancreas volume can be seen, indicating pancreatitis without stones in the biliary tract (Figure 8) [109]. USG or CT can easily detect the presence of these findings.

### 3.6. Nervous system involvement

Approximately one-quarter of patients with COVID-19 have some form of neurological pathology [110]. In hospitalized patients, the rate of neurological complications varies between 6 and 36%, with acute cerebrovascular diseases being the most common and serious [111]. The most common symptoms are altered mental states, focal deficits, and seizures due to ischemic stroke [112]. Other nonspecific clinical symptoms include headache, confusion, and dizziness. Loss of smell and taste in the early stages of COVID-19 disease occurs in approximately 15% of patients [113]. Most patients with neurological involvement have at least one preexisting comorbidity, such as cardiovascular disease and diabetes [112].

Nervous system imaging findings associated with COVID-19 disease can be categorized as follows: cerebrovascular disorder, encephalitis, other encephalopathies, and peripheral nervous system abnormalities. Cerebrovascular disease may refer to the presence of large or small vessel ischemic infarction or hemorrhagic infarction due to venous sinus thrombosis, as well as microhemorrhages involving the juxtacortical and callosal white matter or gross hemorrhage [114]. Encephalitis is diagnosed based on cortical FLAIR signal abnormality regardless of diffusion imaging feature. Although some publications indicate that medial temporal lobe involvement is more common, there is no consensus on this issue [115]. In addition, there may be an abnormal leptomeningeal enhancement in encephalitis, which is sometimes only seen in the postcontrast FLAIR sequence [116]. Depending on encephalitis, differences in signal and enhancement and diffusion abnormalities can also be seen in the basal ganglia, especially the substantia nigra, globus pallidus, and striatonigral pathways [113].

Another form of neurological involvement seen in COVID-19 is diffuse leukoencephalopathy characterized by symmetrical and multifocal hyperintense lesions in the supratentorial white matter and diffusion restriction in which the juxtacortical white matter is partially preserved. Sometimes the lesions can be hemorrhagic [114,116]. These findings, possibly due to hypoxia, are seen in critically ill patients receiving long-term ventilator support and associated with increased mortality [117]. Acute necrotizing encephalopathy, which is included in some case reports and considered to be caused by the cytokine storm, is among the neurological complications. This type of involvement is characterized by rim-shaped symmetrical hemorrhagic lesions in the thalamus, medial temporal lobe, and subinsular region [118]. Widespread brainstem involvement may also be seen. Similar findings have been reported in patients with COVID-19 presenting with hypoxic-ischemic encephalopathy or posterior reversible encephalopathy syndrome [112].

In patients with anosmia, abnormal hyperintensity and enhancement may be seen in the olfactory bulbus and gyrus rectus, despite imaging findings being completely normal in the early stage of the disease [119]. Lastly, in Guillain–Barré syndrome associated with SARS-CoV-2, enhancement can be seen in the caudal nerve roots [120].

## 4. Conclusion

Radiological imaging plays a supportive role in the care of patients with COVID-19. Imaging findings are very diverse, and depending on the stage of the disease, their frequency and distribution vary. Comprehensive studies on the imaging of COVID-19 pneumonia emphasize the importance of CT, although other imaging methods can also be used to diagnose and treat COVID-19 infection. Pulmonary magnetic resonance imaging is not typically considered among the first-line modalities in investigating suspected lower respiratory tract infections, but it may be a viable alternative for patient groups where excessive or repeated exposure to ionizing radiation should be avoided.

COVID-19 pneumonia progresses with bilateral GGOs, primarily distributed in the peripheral parts of the lower lung lobes and often accompanying consolidations on CT scans. However, these findings are not specific and significantly overlap those related to the other causes of viral pneumonia, and alternative diagnoses should be considered in patients with negative diagnostic tests. 

The respiratory tract is the primary system affected by COVID-19, but the cardiovascular system, GIS, hepatobiliary system, GUS, and the nervous system can also show signs and symptoms associated with COVID-19. Therefore, both clinicians and radiologists need to know the extrapulmonary findings and imaging features of COVID-19 to help diagnose and manage the possible complications of the disease in an early stage. They should also be familiar with CT findings that can be seen in patients with COVID-19 since the disease can be incidentally detected during imaging performed with other indications.
